# Metabolic profiling in periparturient dairy cows and its relation with metabolic diseases

**DOI:** 10.1186/s13104-022-06130-z

**Published:** 2022-06-28

**Authors:** Mojahidul Kabir, Md. Mehedi Hasan, Nobonita Sarker Tanni, Mst. Sonia Parvin, Md. Asaduzzaman, Md. Amimul Ehsan, Md. Taohidul Islam

**Affiliations:** 1 Population Medicine and AMR Laboratory, Department of Medicine, Faculty of Veterinary Science, Bangladesh Agricultural University, Mymensingh, 2202 Bangladesh; 2 American Dairy Limited, Sreepur, Gazipur Bangladesh

**Keywords:** Metabolic profiling, Periparturient period, Dairy cows, Metabolic diseases

## Abstract

**Objective:**

Periparturient period is associated with multiple changes including serum concentration of macro minerals and drop in feed intake. Therefore, it is essential to know the actual concentrations of major macro minerals, glucose and ketone bodies in blood during the periparturient period. The objectives of the study were to study the dynamics of calcium, magnesium, phosphorus, and glucose in serum and ketone bodies in the urine of periparturient cows and to estimate the incidence of subclinical metabolic diseases.

**Results:**

Results showed that all the urine samples were negative for ketone bodies. Incidence of subclinical form of hypocalcaemia, hypomagnesaemia, hypophosphatemia, and hypoglycemia in periparturient cows was 31.03%, 48.28%, 17.24% and 55.17%, respectively. Older cows with high BCS and milk yield were mostly affected with a subclinical form of hypocalcaemia and hypoglycemia. No significant differences were observed in serum level of macro minerals and glucose at different time points of periparturient period of cows fed with a balanced ration, and between two groups of cows those were given IV injection of calcium and magnesium on the day of parturition and those were not given. Age, parity, and milk yield had no significant effect on the concentration of calcium, magnesium, phosphorus, and glucose.

**Supplementary Information:**

The online version contains supplementary material available at 10.1186/s13104-022-06130-z.

## Introduction

Periparturient period, particularly the postpartum period is the most vulnerable period for the occurrence of metabolic disorders in dairy cows. Approximately 75% of the diseases in dairy cows typically occur in the first month after calving [1]. Periparturient period or transition period generally extends from 3 weeks prior to parturition through 3 weeks after parturition [2]. This period is associated with multiple changes including hormonal changes, moving from a non-lactating to lactating state as well as a major drop in feed intake [3]. For this reason, serum levels of major macro minerals and glucose are frequently changed in this period [3]. Metabolic diseases are associated with low concentrations of these components in blood. Moreover, subclinical forms of mineral deficiencies have been incriminated for causing a decrease in production levels and decreased feed efficiency, which can cause huge losses to dairy farmers [4]. In this regard, metabolic profiling, which refers to the analysis of blood biochemical constituents, is an important tool for detecting metabolic disorders in dairy cattle before any clinical manifestations appear [5, 6].

Nutritional management in the early dry period is important for maintaining the health and productivity of cows in the transition period [7] as dry matter intake tends to decrease by more than 30% in the last three weeks of gestation [8]. The incidence of metabolic diseases can be reduced by increasing dry matter intake and minimizing the period of negative energy balance after calving [9].

Few studies on metabolic diseases of crossbred lactating cows have been conducted in Bangladesh and reported 30% subclinical hypocalcaemia and 25% subclinical ketosis [10, 11]. No longitudinal studies have so far been conducted on metabolic profiling of blood in dairy cows throughout the periparturient period. Furthermore, it is important to have baseline data on actual serum concentration of macro minerals, glucose, and ketone bodies during periparturient period in relation to the occurrence of subclinical form of metabolic diseases in the context of our country and other countries having similar climatic and management aspects that will certainly be helpful to prevent metabolic diseases. This study, therefore, was conducted (i) to study the dynamics of calcium, magnesium, phosphorus and glucose in serum and ketone bodies in urine of periparturient crossbred cows, and (ii) to estimate the incidence of subclinical form of hypocalcaemia, hypomagnesaemia, hypophosphatemia and hypoglycemia in periparturient crossbred cows in relation to age, parity, body condition score and milk yield.

## Main text

### Methods

#### Study areas and selection of cows

A 6-month prospective cohort study was conducted in an organized dairy cattle farm of Gazipur district. A total of 832 cattle (297 cows, 200 bulls, 235 calves and 100 heifers) were in the farm. A total of 29 pregnant crossbred dairy cows were included in the study based on the availability during study period.

#### Management practices of the farm and cows

The large-scale dairy cattle farm practices zero grazing. The feeding practice was a “cut-and-carry system.” Green grasses provided were mainly Napier, Para, German and seasonal maize. Cows were provided with a varied amount of concentrate in addition to green grasses (Additional file 1: Table S1). Moreover, oral multivitamin-mineral supplementation was given 2–3 days before parturition and throughout the lactation period. On the day of parturition, most of the cows were given IV injection of Cal-D-Mag (Renata Animal Health, Bangladesh), which contains calcium, magnesium, phosphorus, glucose, and boric acid.

#### Data collection

Cow and farm-level data were collected by interviewing the veterinary officer, artificial inseminator, and animal attendant as well as examining the cows and farm. Data were also extracted from farm records.

#### On farm test for ketone bodies in urine

On farm qualitative test for the presence of ketone bodies in urine was performed using commercial reagent strips (Keto-Diastix; Bayer Health Care, Germany) as per the manufacturer’s instruction.

#### Blood sample collection

A total of 203 blood samples were collected from all the selected cows at different occasions (8–14 days, 4–7 days, and 2–3 days before parturition; at the day of parturition; 3rd, 7th and 14th day after parturition). Sera were separated and stored at −20 °C for further analysis*.*

#### Biochemical analysis

Sera samples were tested to measure the concentration of calcium, magnesium, phosphorus, and glucose by using commercially available reagents (for glucose–JTC Diagnostics, Germany; for calcium, magnesium and phosphorus–Linear Chemicals, Spain), and the absorbance values of samples and standard were recorded by using UV spectrophotometer.

Concentration was calculated by the following formula:

$$\frac{{\text{Absorbacne of sample}}}{{\text{Absorbance of standard}}} \times {\text{Standard concentration}} = {\text{Concentration in mg/dL}}$$

## Diagnosis of subclinical form of hypocalcaemia, hypomagnesaemia, hypophosphatemia and hypoglycemia

Cows having serum concentration of calcium < 8–6 mg/dL, magnesium < 1.8–1.1 mg/dL, phosphorus < 4–2 mg/dL and glucose < 40–25 mg/dL, were diagnosed as subclinically hypocalcaemic, hypomagnesaemic, hypophosphatemic and hypoglycemic, respectively [12–14].

### Statistical analysis

The repeated measures ANOVA and independent samples t-test were performed to find out the significant differences in mean serum levels of macro minerals and glucose at different occasions and in relation to cows’ factors within and between groups, respectively. The Z-test for proportions was performed to analyze the percent values. The SPSS version 22.0 was used for the analyses.

## Results

### Ketone bodies in urine

All the urine samples were found negative for the presence of ketone bodies indicating that all the samples had the level of ketone bodies < 5 mg/dL.

### Serum concentration of macro minerals and glucose

**Fig. 1:**
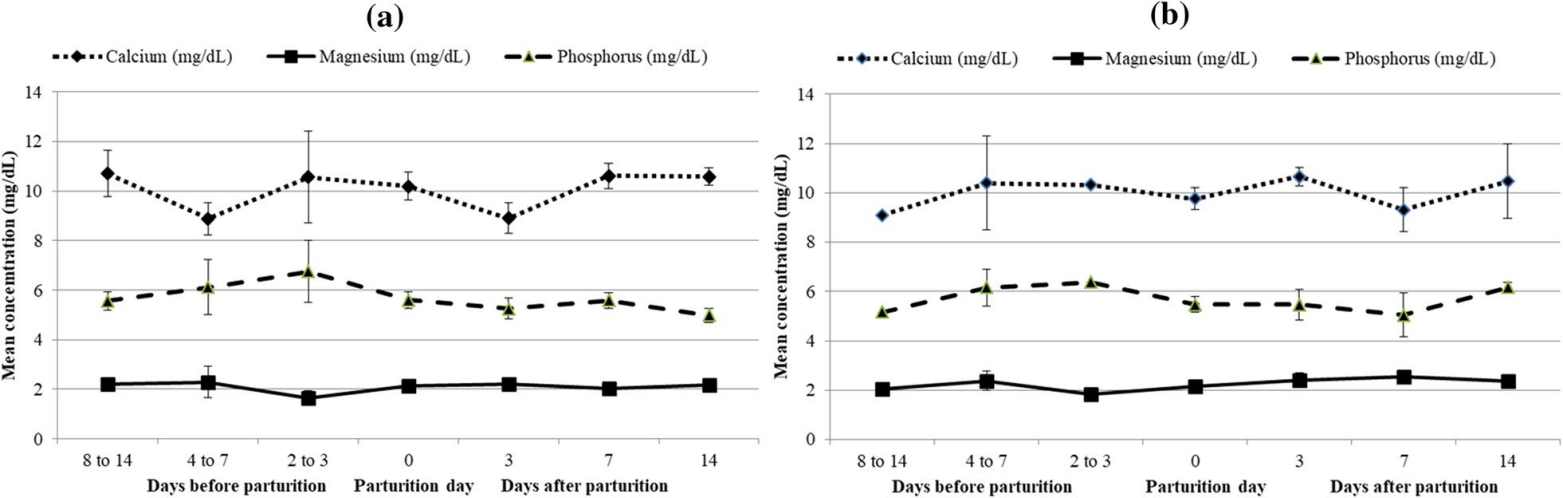
Line graph showing non-significant variation in mean serum concentration of Ca, Mg & P in periparturient cows those **a** received IV injection of Cal-D-Mag (Renata Animal Health) and **b** were not received the same at the day of parturition. [Cal-D-Mag (Renata Animal Health) contains Ca, Mg, P & glucose]

**Fig. 2:**
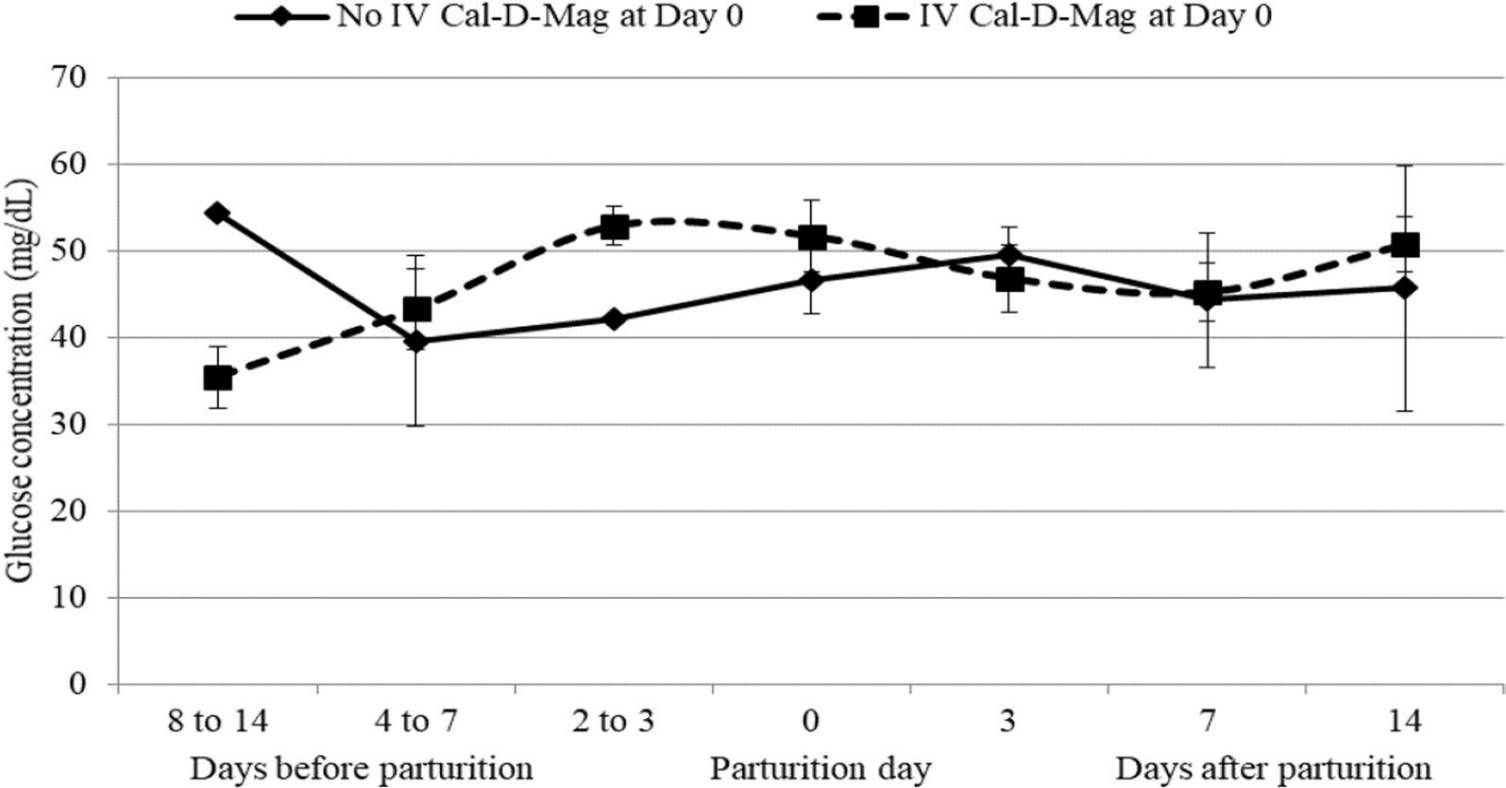
Serum glucose concentration in periparturient cows

The serum levels (mg/dL) of Ca, Mg, P and glucose before vs after parturition ranged from 9.09 to 10.71 vs 8.91 to 10.58, 1.64 to 2.38 vs 2.03 to 2.56, 5.17 to 6.76 vs 4.99 to 6.17, and 35.49 to 52.94 vs 44.34 to 50.78 irrespective of IV injection of Cal-D-Mag (contains calcium, magnesium, phosphorus, glucose and boric acid) at day 0 of parturition (Additional file 1: Table S2, Figs. 1, 2). No significant variation was observed in serum level of these elements at different occasions from 14 days before to 14 days after parturition. However, serum level of calcium and phosphorus decreased from 2 to 3 days before parturition to the day of parturition. Furthermore, IV injection of Cal-D-Mag at day 0 of parturition had no significant effect on serum levels of these elements.

No significant differences were observed in mean calcium, magnesium, phosphorus, and glucose concentrations due to differences in age, parity, and milk yield (Additional file 1: Table S3). However, a significant difference (*P* ≤ 0.05) in magnesium concentration was observed between cows with BCS 3 to < 4 and BCS ≥ 4.

### Incidence of subclinical form of hypocalcaemia, hypomagnesaemia, hypophosphatemia and hypoglycemia

The overall incidence of subclinical form of hypocalcaemia, hypomagnesaemia, hypophosphatemia and hypoglycemia in periparturient crossbred cows was 31.03%, 48.28%, 17.24% and 55.17%, respectively (Table 1). Among the hypocalcaemic cows, 33.33% cows had subclinical form of hypophosphatemia and 55.55% cows were subclinically hypomagnesaemic. Both subclinical hypomagnesaemia and subclinical hypophosphatemia found in 22.22% subclinically hypocalcaemic cows.

**Table 1:** Incidence of subclinical form of hypocalcaemia, hypomagnesaemia, hypophosphatemia and hypoglycemia in periparturient crossbred cows according to age, parity, BCS and milk yield

Variables		Subclinical Hypocalca-emia	Subclinical Hypomagn-esaemia	Subclinical Hypophos-phatemia	Subclinical Hypoglyc-emia
Age (years)	2 to 4 (n = 19)	6 (31.58%)^a^	7 (36.84%)^a^	2 (10.53%)^a^	12 (63.18%)^a^
	> 4 to 6 (n = 7)	2 (28.57%)^a^	5 (71.43%)^a^	2 (28.57%)^a^	4 (57.14%)^ab^
	> 6 (n = 3)	1 (33.33%)^a^	2 (66.67%)^a^	1 (33.33%)^a^	0 (0.00%)
Parity	1 (n = 6)	1 (16.67%)^a^	3 (50.00%)^a^	0 (0.00%)^a^	3 (50.00%)^ab^
	2 (n = 14)	6 (42.86%)^a^	5 (35.71%)^a^	3 (21.43%)^a^	9 (64.29%)^b^
	3 (n = 6)	1 (16.67%)^a^	4 (66.67%)^a^	1 (16.67%)^a^	4 (66.67%)^ab^
	≥ 4 (n = 3)	1 (33.33%)^a^	2 (66.67%)^a^	1 (33.33%)^a^	0 (0.00%)
BCS	< 3 (n = 2)	0 (0.00%)	0 (0.00%)	0 (0.00%)	1 (50.00%)^a^
	3 to < 4 (n = 19)	6 (31.58%)^a^	12 (63.16%)^a^	3 (15.79%)^a^	10 (52.63%)^a^
	≥ 4 (n = 8)	3 (37.50%)^a^	2 (25.00%)^a^	2 (25.00%)^a^	5 (62.50%)^a^
Milk yield (L)	5 to 10 (n = 6)	0 (0.00%)	1 (16.67%)^a^	1 (16.67%)^a^	3 (50.00%)^a^
	> 10 to 15 (n = 7)	3 (42.86%)^ab^	6 (85.71%)^b^	1 (14.29%)^a^	3 (42.86%)^a^
	> 15 to 20 (n = 10)	3 (30.00%)^ab^	7 (70.00%)^b^	2 (20.00%)^a^	6 (60.00%)^a^
	> 20 (n = 6)	3 (50.00%)^b^	0 (0.00%)	1 (16.67%)^a^	4 (66.67%)^a^
Overall (n = 29)		9 (31.03%)	14 (48.28%)	5 (17.24%)	16 (55.17%)

Values bearing different superscripts within the column for each variable differ significantly (P ≤ 0.05)

Cows with a daily milk yield > 20 L had a 50% higher incidence of subclinical hypocalcaemia. Cows producing > 10–20 L of milk per day had a significantly (*P* ≤ 0.05) higher incidence of subclinical hypomagnesaemia (70–86%) than cows producing 5–10 L of milk per day (17%) (Table 1).

Here in this study, we did not observe any significant effect of age, BCS and parity of cows on the occurrence of subclinical hypocalcaemia, hypomagnesaemia and hypophosphatemia. Farm records revealed that, there were no occurrences of clinical cases of hypocalcaemia, hypomagnesaemia and ketosis.

## Discussion

Wide hematological, physiological and biochemical changes occur in dairy cows around the periparturient period. The present study reports the changes in concentration of calcium, magnesium, phosphorus and glucose in serum and ketone bodies in urine as well as incidence of subclinical form of hypocalcaemia, hypomagnesaemia, hypophosphatemia and hypoglycemia in periparturient dairy cows. In this study, the serum levels (mg/dL) of Ca, Mg, P and glucose in periparturient cows varied from 8.91 to 10.58, 1.64 to 2.56, 4.99 to 6.76, and 35.49 to 52.94, respectively. The serum level of calcium and phosphorus decreased from 2 to 3 days before parturition to the day parturition. Preliminary studies on the metabolic profile of dairy cows revealed that there was decrease in blood calcium and phosphorus at the day of parturition [15–17]. A previous study reported that concentration of calcium, magnesium, phosphorus and glucose in serum ranges from 6.05 to 11.98 mg/dL, 1.43 to 4.25 mg/dL, 2.47 to 6.99 mg/dL and 20.83 to 84.54 mg/dL, respectively [11].

No significant differences observed in serum level of glucose and macro minerals at different occasions 14 days before to 14 days after parturition and in between two groups of cows those were given IV injection of Cal-D-Mag (contains calcium, magnesium, phosphorus and boric acid) at the day of parturition and those were not given. This may be due to proper feeding and management practices adopted in the farm. No significant differences observed in concentration of calcium, magnesium, phosphorus and glucose due to age, parity and milk yield. Previous finding also reported that, in clinically normal cows, variation of these blood parameters due to age, parity and milk yield is limited by homeostatic control systems [18].

The incidence of subclinical form of hypocalcaemia, hypomagnesaemia, hypophosphatemia and hypoglycemia was 31.03%, 48.28%, 17.24% and 55.17%, respectively which is consistent with earlier reports on the occurrence of subclinical hypocalcaemia and subclinical ketosis in prepaturient and lactating cows that ranges from 25 to 54% and 12 to 47% [10, 11, 14, 19–21].

The present study clearly demonstrates that incidence of subclinical hypocalcaemia and subclinical hypoglycemia was higher in cows with high BCS ≥ 4 and milk production > 20 L. Previous studies also reported higher occurrence of subclinical form of hypocalcaemia and ketosis in older cows with high milk production [14, 22–28]. Cows that are obese at calving tend to have higher risk of metabolic disorder because of excessive mobilization of body fat reserves [29, 30]. Earlier report also stated that the cows with pre-partum BCS ≥ 3.75 were 5.25 times more at risk of developing subclinical ketosis than cows with pre-partum BCS ≤ 3.5 [31]. High milk production results in losses of more Ca and other minerals with milk which predispose to various metabolic diseases [32, 33].

Subclinical hypocalcaemia and subclinical hypophosphatemia were more common in cows more than 6 years old. The finding is also in agreement with previous reports. The incidence of metabolic diseases increases with the age of cow [3, 12, 14, 27]. Milk production increases with age, which results in higher demand for calcium. But, the ability to mobilize calcium from bone stores also declined with age [14, 34]. Moreover, intestinal receptors for 1,25(OH)2D3 decline in older cows which impair the active transport of calcium in the intestine [14, 32, 34].

Although subclinical metabolic diseases were found, no cows were observed to develop clinical form of disease. It might be due to rearing of cows with proper management and supply of required amount of roughage and properly mixed concentrates. Balanced ration was provided to periparturient cows which contained calcium, magnesium, phosphorus and carbohydrate rich ingredients, and multivitamin-minerals supplementation was also added to the ration. Previous finding also reported that proper nutrition and management results in low incidence of periparturient diseases [35].

## Conclusion

Periparturient period is the most vulnerable period for the occurrence of metabolic diseases. Older cows with high BCS and milk yield are mostly affected with subclinical form of hypocalcaemia and hypoglycemia. However, the risk of metabolic diseases in periparturient dairy cows can be reduced by providing balanced ration according to stage of pregnancy and lactation and oral supplementation of multivitamin-minerals from 2 to 3 days before parturition and throughout the lactation period. The IV injection of Cal-D-Mag (preparation of Ca, Mg, P and glucose) at the day of parturition has no significant effect on serum concentration of calcium, magnesium, phosphorus and glucose if balanced ration is provided to cows.

## Limitations

It would be worthwhile if we could sample more cows. However, all the pregnant cows in the farm during the study period were included.

## Supplementary Information

**Additional file 1:** **Table S1.** Ration (concentrated feed) formulation chart for dry cows, pregnant cows and lactating cows of the farm. **Table S2.** Serum concentration (Mean±SE) of macro minerals and glucose in periparturient crossbred cows in an organized dairy farm in Gazipur district. **Table S3.** Serum concentration (Mean±SE) of macro minerals and glucose according to age, parity, BCS and milk yield in periparturient crossbred cows in an organized dairy farm in Gazipur district.

## Data Availability

All the data are presented in the manuscript in summarized form. However, raw data may be provided upon request from the corresponding author.

## Abbreviations

Ca: Calcium
Mg: Magnesium
P: Phosphorus
Kg: Kilograms
IV: Intravenous
mL: Millilitre
rpm: Revolutions per minute
UV: Ultraviolet
SPSS: Statistical Package for Social Services
ANOVA: Analysis of variance
BCS: Body condition scoring
