# Supplementary material for: Metabolic profiling in periparturient dairy cows and its relation with metabolic diseases
Source: BMC Res Notes. 2022 Jun 28;15:231. doi: 10.1186/s13104-022-06130-z (PMC9238095; doi:10.1186/s13104-022-06130-z)
Supplement: Supplementary file 1 — Additional file 1: Table S1. Ration (concentrated feed) formulation chart for dry cows, pregnant cows and lactating cows of the farm. Table S2. Serum concentration (Mean±SE) of macro minerals and glucose in periparturient crossbred cows in an organized dairy farm in Gazipur district. Table S3. Serum concentration (Mean±SE) of macro minerals and glucose according to age, parity, BCS and milk yield in periparturient crossbred cows in an organized dairy farm in Gazipur district. [file 13104_2022_6130_MOESM1_ESM.docx]

**Additional Materials - Shihab *et al.***

**Table S1** Ration (concentrated feed) formulation chart for dry cows, pregnant cows and lactating cows of the farm

| SN | Ingredients | Dry cows  (%) | Pregnant cows (%) | Lactating cows (%) |
| --- | --- | --- | --- | --- |
| 1 | Wheat bran | 56 | **50** | 44 |
| 2 | Soybean meal | 16 | 24 | 30 |
| 3 | Maize | 12 | 12 | 12 |
| 4 | Rice husk | 12 | 10 | 10 |
| 5 | Dolomite | 1 | 1 | 1 |
| 6 | Limestone | 2.475 | 2.475 | 2.475 |
| 7 | Rock salt | 0.4 | 0.4 | 0.4 |
| 8 | Blackjack | 0.1 | 0.1 | 0.1 |
| 9 | Yeast | 0.025 | 0.025 | 0.025 |
| Total | | 100 | 100 | 100 |
| Daily amount for each cow | | 4 kg | 6 kg  (8 kg in last  3 weeks of pregnancy) | 10 kg |

**Table S2** Serum concentration (Mean±SE) of macro minerals and glucose in periparturient crossbred cows in an organized dairy farm in Gazipur district

| Parameter | IV injection of Ca, Mg, P & glucose at the day of parturition | Days before parturition | | | Day  0 | Days after parturition | | |
| --- | --- | --- | --- | --- | --- | --- | --- | --- |
|  |  | 8 to 14 | 4 to 7 | 2 to 3 |  | 3 | 7 | 14 |
| Calcium (mg/dL) | Yes  (n = 22) | 10.71^a^  ±0.93 | 8.88^a^  ±0.64 | 10.57^a^  ±1.85 | 10.19^a^  ±0.57 | 8.91^a^  ±0.61 | 10.62^a^  ±0.51 | 10.58^a^  ±0.36 |
|  | No  (n = 7) | 9.09^a^  ±0.00 | 10.40^a^  ±1.89 | 10.32^a^  ±0.00 | 9.75^a^  ±0.45 | 10.65^a^  ±0.37 | 9.31^a^  ±0.89 | 10.47^a^  ±1.52 |
| Magnesium (mg/dL) | Yes  (n = 22) | 2.21^a^  ±0.13 | 2.29^a^  ±0.64 | 1.64^a^  ±0.30 | 2.15^a^  ±0.11 | 2.21^a^  ±0.13 | 2.03^a^  ±0.13 | 2.19^a^  ±0.11 |
|  | No  (n = 7) | 2.06^a^  ±0.00 | 2.38^a^  ±0.38 | 1.83^a^  ±0.00 | 2.16^a^  ±0.14 | 2.41^a^  ±0.28 | 2.56^a^  ±0.23 | 2.38^a^  ±0.21 |
| Phosphorus (mg/dL) | Yes  (n = 22) | 5.57^a^  ±0.37 | 6.11^a^  ±1.11 | 6.76^a^  ±1.24 | 5.60^a^  ±0.34 | 5.26^a^  ±0.42 | 5.58^a^  ±0.32 | 4.99^a^  ±0.28 |
|  | No  (n = 7) | 5.17^a^  ±0.00 | 6.16^a^  ±0.74 | 6.37^a^  ±0.00 | 5.47^a^  ±0.31 | 5.46^a^  ±0.62 | 5.05^a^  ±0.89 | 6.17^a^  ±0.18 |
| Glucose (mg/dL) | Yes  (n = 22) | 35.49^a^  ±3.52 | 43.32^a^  ±4.69 | 52.94^a^  ±2.23 | 51.76^a^  ±4.18 | 46.87^a^  ±3.89 | 45.27^a^  ±3.43 | 50.78^a^  ±3.17 |
|  | No  (n = 7) | 54.39^a^  ±0.00 | 39.64^a^  ±9.82 | 42.24^a^  ±0.00 | 46.68^a^  ±3.92 | 49.58^a^  ±3.19 | 44.34^a^  ±7.73 | 45.77^a^  ±14.18 |

Values bearing same superscript within the column for each parameter as well as within the row did not differ significantly (P>0.05).

Reference values of normal blood calcium 8.4-10.8 mg/dL, magnesium 1.94-3.16 mg/dL, phosphorus 4-8.76 mg/dL and glucose 40-88 mg/dL (Goff, 2006a; Yameogo et al., 2008; Quiroz-Rocha et al., 2009; Zhang et al., 2009).

**Table S3** Serum concentration (Mean±SE) of macro minerals and glucose according to age, parity, BCS and milk yield in periparturient crossbred cows in an organized dairy farm in Gazipur district

| Parameters | | Calcium (mg/dL) | Magnesium (mg/dL) | Phosphorus (mg/dL) | Glucose  (mg/dL) |
| --- | --- | --- | --- | --- | --- |
| Age  (years) | 2 to 4 (n =19) | 10.07±0.38^a^ | 2.16±0.06^a^ | 5.41±0.14^a^ | 45.82±1.92^a^ |
|  | >4 to 6 (n =7) | 10.29±0.38^a^ | 2.17±0.09^a^ | 5.92±0.29^a^ | 47.74±2.18^a^ |
|  | >6 (n =3) | 10.22±0.50^a^ | 2.25±0.18^a^ | 5.50±0.38^a^ | 50.94±3.12^a^ |
| Parity | 1 (n =6) | 10.05±0.51^a^ | 1.95±0.11^a^ | 5.37±0.27^a^ | 52.55±4.07^a^ |
|  | 2 (n =14) | 9.10±0.29^a^ | 2.23±0.06^a^ | 5.33±0.16^a^ | 44.66±2.05^a^ |
|  | 3 (n =6) | 10.45±0.42^a^ | 2.18±0.1^a^ | 6.13±0.31^a^ | 45.84±2.08^a^ |
|  | ≥4 (n =3) | 10.22±0.50^a^ | 2.24±0.18^a^ | 5.50±0.38^a^ | 50.94±3.12^a^ |
| BCS | <3 (n =2) | 9.63±0.71^a^ | 2.16±0.07^a^ | 5.17±0.18^a^ | 39.30±4.33^a^ |
|  | 3 to <4 (n =19) | 9.97±0.23^a^ | 2.08±0.06^ab^ | 5.54±0.18^a^ | 47.35±1.71^a^ |
|  | ≥4 (n =8) | 10.58±0.41^a^ | 2.34±0.08^a^ | 5.74±0.23^a^ | 47.30±2.47^a^ |
| Milk Yield (L) | 5 to 10 (n =6) | 10.72±0.50^a^ | 2.18±0.17^a^ | 5.26±0.25^a^ | 52.57±4.41^a^ |
|  | >10 to 15 (n =7) | 10.11±0.48^a^ | 1.99±0.11^a^ | 5.48±0.30^a^ | 52.72±3.17^a^ |
|  | >15 to 20(n =10) | 9.83±0.30^a^ | 2.14±0.08^a^ | 5.64±0.26^a^ | 44.49±2.27^a^ |
|  | >20(n =6) | 10.30±0.55^a^ | 2.40±0.08^a^ | 5.39±0.21^a^ | 44.71±2.99^a^ |

Values bearing different superscript within the column for each parameter differ significantly (*P*≤0.05).
